# Iron chelation inhibits mTORC1 signaling involving activation of AMPK and REDD1/Bnip3 pathways

**DOI:** 10.1038/s41388-020-1366-5

**Published:** 2020-06-15

**Authors:** Chaowei Shang, Hongyu Zhou, Wang Liu, Tao Shen, Yan Luo, Shile Huang

**Affiliations:** 1 Department of Biochemistry and Molecular Biology, Louisiana State University Health Sciences Center, Shreveport, LA 71130-3932 USA; 2 Feist-Weiller Cancer Center, Louisiana State University Health Sciences Center, Shreveport, LA 71130-3932 USA

**Keywords:** Stress signalling, Target identification

## Abstract

The mammalian target of rapamycin (mTOR) functions as two complexes (mTORC1 and mTORC2), regulating cell growth and metabolism. Aberrant mTOR signaling occurs frequently in cancers, so mTOR has become an attractive target for cancer therapy. Iron chelators have emerged as promising anticancer agents. However, the mechanisms underlying the anticancer action of iron chelation are not fully understood. Particularly, reports on the effects of iron chelation on mTOR complexes are inconsistent or controversial. Here, we found that iron chelators consistently inhibited mTORC1 signaling, which was blocked by pretreatment with ferrous sulfate. Mechanistically, iron chelation-induced mTORC1 inhibition was not related to ROS induction, copper chelation, or PP2A activation. Instead, activation of AMPK pathway mainly and activation of both HIF-1/REDD1 and Bnip3 pathways partially contribute to iron chelation-induced mTORC1 inhibition. Our findings indicate that iron chelation inhibits mTORC1 via multiple pathways and iron is essential for mTORC1 activation.

## Introduction

The mechanistic/mammalian target of rapamycin (mTOR), a serine/threonine kinase, plays critical roles in regulating cell growth, proliferation, survival, and motility through sensing environmental cues. mTOR functions as two complexes, mTORC1 and mTORC2 [1, 2]. mTORC1 contains mTOR, mLST8 (mammalian let, raptor (regulatory-associated protein of mTOR) [3–5]), and PRAS40 (proline-rich Akt substrate 40). PRAS40 negatively regulates mTORC1 through interaction with mTOR and raptor, which hinders the recruitment of mTORC1 substrates [6–8].

Activation of mTORC1 promotes anabolic processes involved in cell growth and metabolism. mTORC1 phosphorylates S6K1 (p70 S6 kinase 1) and 4E-BP1 (eukaryotic translation initiation factor 4E-binding protein 1), promoting protein/lipid/nucleotide synthesis and cell growth [9, 10]. Aberrant activation of mTOR signaling is frequently observed in various tumors due to mutations of genes (e.g. *PTEN, PIK3CA, LKB1*, and *TSC1/2*) [1, 2]. Thus, enormous efforts have been made to develop mTOR inhibitors for targeted cancer therapy.

mTORC1 is comprehensively regulated by multiple pathways. It lies downstream of the PI3K/Akt pathway. When the PI3K/Akt pathway is activated, Akt can phosphorylate TSC2 (tuberous sclerosis complex 2) and interrupt the integrity of TSC1/2 complex [11–14]. The TSC1/2 complex antagonizes Rheb (ras homolog enriched in brain), thereby inhibiting Rheb-mediated mTORC1. Akt-induced TSC1/2 dissociation impedes the negative regulation of TSC1/2 on Rheb, hence activating mTORC1 [15–18]. Akt also activates mTORC1 through phosphorylating and releasing PRAS40 from mTORC1 [6–8].

TSC1/2 activity is also regulated by REDD1 (regulated in development and DNA damage response 1), a target of transcription factor HIF-1 (hypoxia-inducible factor 1) [19]. Under hypoxic conditions, HIF-1 activates REDD1, which then promotes activation of TSC1/2 [20]. Activated TSC1/2 further inhibits mTORC1. Besides, REDD1 can also bind to Akt and PP2A (protein phosphatase 2A), leading to inhibition of Akt, thereby inhibiting mTORC1 [21]. Furthermore, Bnip3 (BCl2/adenovirus E1B 19 kDa protein-interacting protein) can be regulated by HIF-1 and inhibit mTORC1 through direct inhibition of Rheb [22]. Moreover, under energy stress, AMPK (AMP-activated protein kinase) is activated, which phosphorylates raptor on S792, inhibiting the activity of mTORC1 [23]. AMPK also inhibits mTORC1 through phosphorylating TSC2 and activating the TSC1/2 complex [24].

Iron is an essential element involved in many human physiological processes [25]. Iron chelators, such as deferoxamine (DFO, also Desferal) and deferasirox (Exjade), have been used to treat iron overload diseases [26]. Epidemiological evidence suggests that excessive iron contributes to tumor initiation, growth, microenvironment, and metastasis [27]. Studies have found that expression levels of iron regulating proteins, such as transferrin receptor are altered in cancer cells, leading to increased intracellular iron level [28–30]. Thus, iron chelators have been investigated as anticancer agents [31].

DFO was first tested in humans to treat leukemia and neuroblastoma [32, 33], but with poor cell membrane permeability [26]. Recently, iron chelators with high cell permeability have been identified and designed, such as CPX (ciclopirox olamine) and Dp44mT [34, 35]. CPX is an off-patent antifungal drug for topical use [36], and Dp44mT is a new iron chelator with high cell membrane permeability [37]. The anticancer effect of iron chelation is closely related to intracellular iron level decrease, induction of ROS (reactive oxygen species), cell cycle arrest, cell death, and tumor suppressor gene expression [34, 38–42]. However, the molecular mechanisms underlying iron chelation-induced anticancer effect are not fully understood. Apart from the inhibition of Wnt/β-catenin signaling pathway and the alteration in the MAPK pathway [43–45], little is known about the effect of iron chelation on other signaling pathways in cancer cells. mTOR responds to oxygen, energy, and oxidative stresses [1, 2], which are associated with intracellular iron level [25, 26]. This study was set to determine whether and how iron chelation inhibits mTOR signaling.

## Results

### Iron chelation selectively inhibits proliferation of cancer cells

**Fig. 1:**
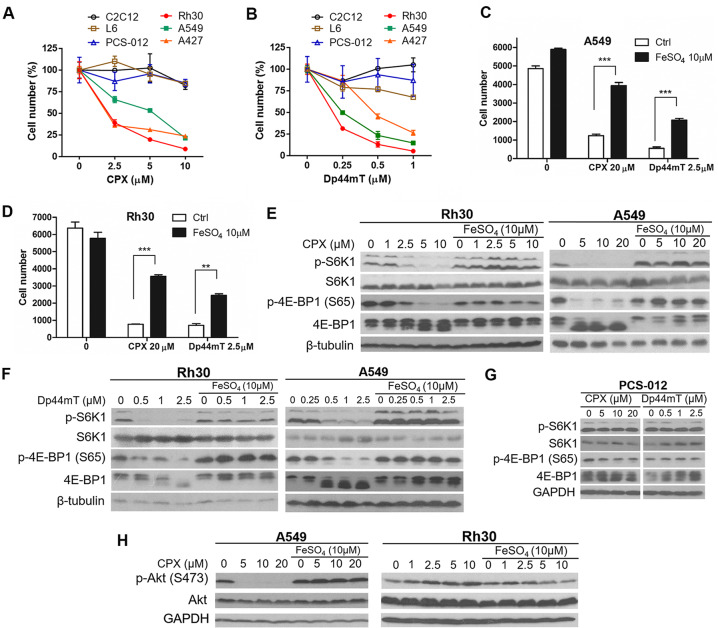
Iron chelation inhibits mTORC1 in cancer cells, but the effect of iron chelation on mTORC2 is cell line dependent. Indicated cell lines were treated with CPX (0–10 µM) (**a**), Dp44mT (0–1 µM) (**b**), or pretreated with/without FeSO4 (10 µM) for 1 h, and then treated with CPX (20 μM) or Dp44mT (2.5 μM) (**c**, **d**) for 48 h, followed by cell counting. Statistical analysis was performed by two-way ANOVA, followed by Bonferroni post-test analysis (*n* = 3) (**P < 0.01, ***P < 0.001). **e**–**h** Indicated cells were pretreated with/without FeSO_4_ (10 µM) for 1 h, and then treated with CPX (0–10 μM or 0–20 μM) or Dp44mT (0–2.5 μM) for 24 h, followed by western blotting with indicated antibodies. Similar results were observed in at least three independent experiments. See also Figs. S1 and S2.

To test whether the anti-proliferative effect of iron chelation is tumor-selective, human rhabdomyosarcoma (Rh30) and lung cancer cells (A549 and A427) were compared with normal human primary fibroblasts (PCS-201-012), mouse myoblasts (C2C12), and rat myoblasts (L6). Cells were treated with CPX or Dp44mT for 48 h, followed by cell counting (Fig. 1a, b). The results showed that all cancer cell lines were sensitive to CPX and Dp44mT (with IC_50_ values of 2–6 μM for CPX; 0.2–0.5 μM for Dp44mT), but all normal cell lines were highly resistant to the two compounds (with IC_50_ values of >10 μM for CPX; > 1 μM for Dp44mT) (Fig. 1a, b). Furthermore, we demonstrated that the anti-proliferative effect was related to iron depletion, as pretreatment with 10 µM of FeSO_4_ for 1 h dramatically rescued the proliferation inhibition (Fig. 1c, d). The results support the notion that cancer cells are addictive to iron for rapid proliferation, and iron chelation has a great potential for tumor-selective treatment.

### Iron chelation inhibits mTORC1 consistently, but can inhibit or activate mTORC2 in a cell line-dependent manner

To study the effect of iron chelation on mTORC1 signaling, Rh30, A549 and A427 cells were pretreated with/without FeSO_4_, followed by treatment with CPX or Dp44mT. Both CPX and Dp44mT inhibited p-S6K1 and p-4E-BP1 in a concentration-dependent manner, which was blocked by pretreatment with FeSO_4_ (Figs. 1e, f and S1A, B), suggesting that iron is essential for mTORC1 activity. Similar results were observed in other cancer cell lines (Rh30, Rh1, RD, and MDA-MB-231) treated with DFO (Fig. S1C–F). Interestingly, in line with the anti-proliferative effect (Fig. 1a, b), iron chelation did not obviously affect p-S6K1 and p-4E-BP1 in normal cells (PCS-201-012, C2C12 and L6) (Figs. 1g and S1G, H). The results support that mTORC1 is more sensitive to iron chelation in cancer cells than in normal cells.

In addition, we also tested the effect of iron chelation on mTORC2. As p-Akt (S473) is often used as a surrogate of mTORC2 activity [46], we examined the level of p-Akt (S473) in cells following 24-h CPX or Dp44mT treatment with/without FeSO_4_ pretreatment. The results showed that in A549 and A427 cells, iron chelation reduced p-Akt (S473) levels (Figs. 1h and S2A), but in Rh30, RD (rhabdomyosarcoma), and HT29 (colon cancer) cells, iron chelation increased p-Akt (S473) levels (Figs. 1h and S2B, C). Pretreatment with FeSO_4_ blocked the inhibitory or stimulatory effects of iron chelators on Akt S473 phosphorylation in all cases tested (Figs. 1h and S2A), suggesting that although iron chelation shows opposite effects on p-Akt (S473) in various cell lines, these effects are still related to the chelation of intracellular iron. In addition, similar pattern was observed in Akt-T308 phosphorylation (Fig. S2D–F). Collectively, these results indicate that iron chelation inhibits mTORC1 consistently, but may inhibit or activate mTORC2 cell line dependently.

### The effect of iron chelation on mTORC1 inhibition is not related to ROS production, copper chelation, or PP2A activation

**Fig. 2:**
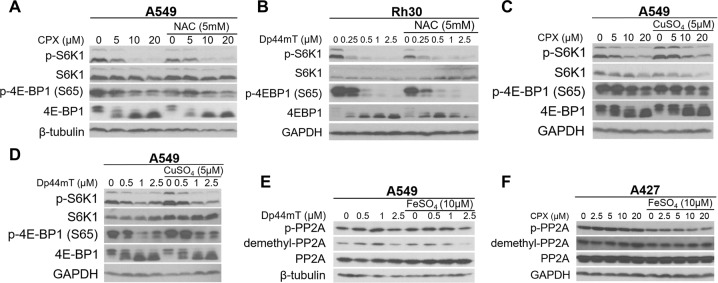
The effect of iron chelation on mTORC1 inhibition is not related to ROS production, copper chelation, or PP2A activation. Indicated cells were pretreated with/without NAC (5 mM) (**a**, **b**), CuSO_4_ (5 μM) (**c**, **d**), or FeSO_4_ (10 µM) (**e**, **f**) for 1 h, and then treated with CPX (0–20 µM) or Dp44mT (0–2.5 µM) for 24 h. Cell lysates (**a**–**f**) were subjected to western blotting with indicated antibodies. Similar results were observed in at least three independent experiments. See also Fig. S3.

mTOR can be inhibited or activated in response to certain oxidative stresses depending on experimental conditions and cell lines [47]. CPX and Dp44mT have ROS generating feature [48, 49]. To determine whether iron chelation-induced mTORC1 inhibition is the consequence of ROS induction, *N*-acetyl-L-cysteine (NAC), a general ROS scavenger and antioxidant, was used. NAC pretreatment did not markedly attenuate iron chelation-induced dephosphorylation of S6K1 and 4E-BP1 in the cells (Fig. 2a, b). The results suggest that iron chelation-induced mTORC1 inhibition should not be associated with ROS induction.

Dp44mT is also capable of chelating copper [49]. To test whether Dp44mT- or CPX-induced mTORC1 inhibition links to copper chelation, A549 cells were pretreated with/without CuSO_4_ (5 µM) for 1 h, followed by 24-h CPX or Dp44mT treatment. CuSO_4_ pretreatment did not obviously affect CPX- or Dp44mT-induced dephosphorylation of S6K1 and 4E-BP1 (Fig. 2c, d), suggesting that the accumulation/chelation of copper is less likely involved in CPX- and Dp44mT-induced mTORC1 inhibition.

It has been shown that DFO inhibits mTORC1 in colon cancer cells partly through activation of PP2A [50]. To determine whether iron chelation inhibits mTORC1 by activating PP2A, we examined the protein levels of p-PP2A (Y307) and demethyl-PP2A, two indicators for PP2A inhibition [51, 52]. The results showed that treatment with CPX or Dp44mT did not decrease the level of p-PP2A (Figs. 2e, f and S3A, B), suggesting no activation of PP2A. Instead, increased p-PP2A was observed in Dp44mT-treated Rh30 cells (Fig. S3B), indicating inhibition of PP2A. Similarly, CPX or Dp44mT did not decrease demethylated-PP2A level either. Noticeably, Dp44mT, at 0.5–1 μM, increased demethylated-PP2A level (Fig. 2e), also indicating inhibition of PP2A. Thus, our data do not support that iron chelation-induced mTORC1 inhibition is due to PP2A activation.

### HIF-1/REDD1 induction is partially involved in iron chelation-induced mTORC1 inhibition

**Fig. 3:**
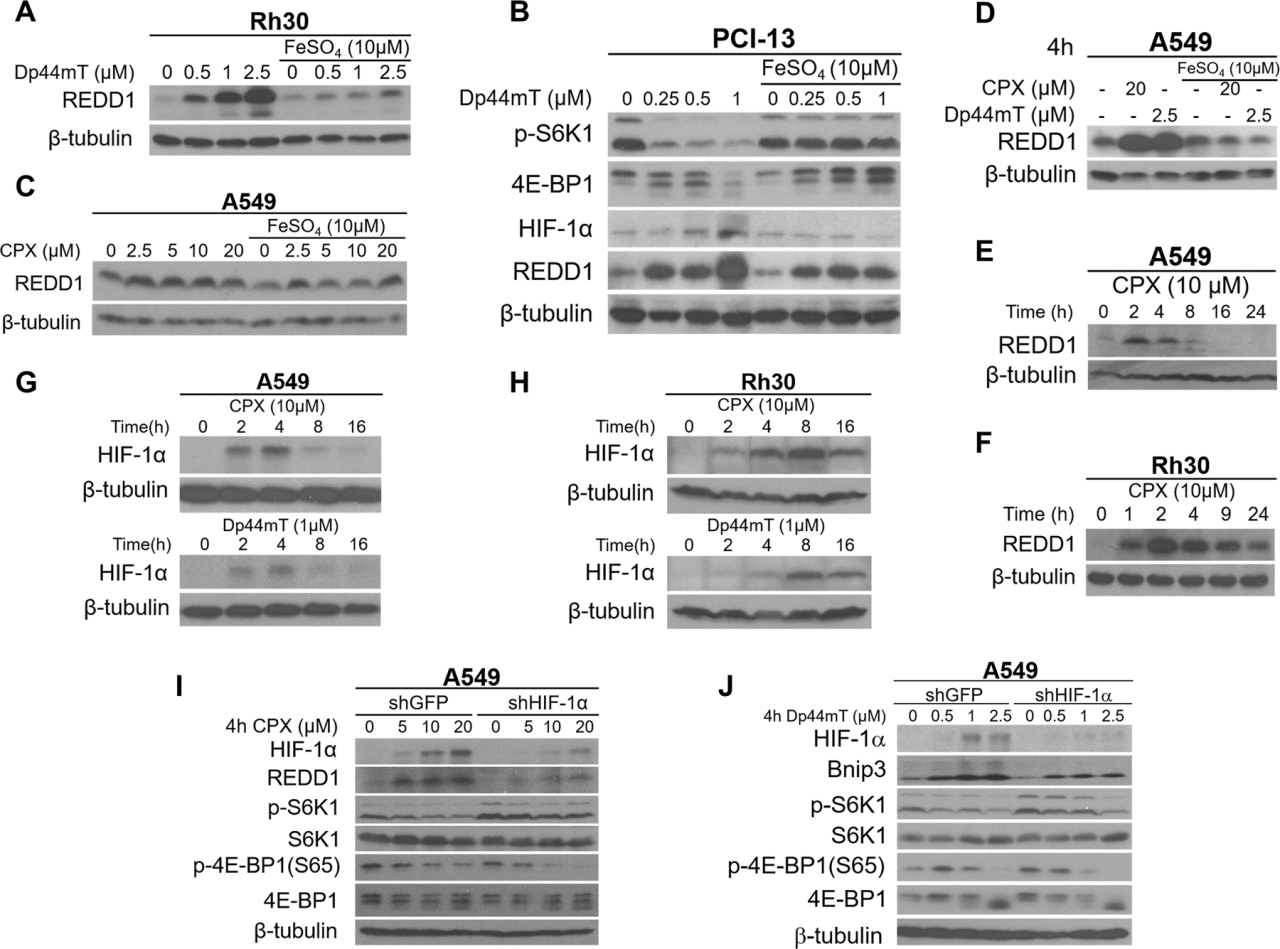
HIF-1/REDD1 pathway transiently and partially mediates iron chelation-induced mTORC1 inhibition. Rh30, PCI-13, and A549 cells were pretreated with/without FeSO_4_ (10 µM) for 1 h, and then treated with CPX (0–20 µM) or Dp44mT (0–2.5 µM) for 24 h (**a**–**c**) or 4 h (**d**). **e**–**h** Indicated cells were treated with CPX (10 µM) or Dp44mT (1 μM) for indicated time. (**i** and **j**) A549 cells, infected with lentiviral shRNA to HIF-1α or GFP (control), were treated with CPX (0–20 µM) or Dp44mT (0–2.5 µM) for 4 h. Cell lysates (**a**–**j**) were subjected to western blotting with indicated antibodies. Similar results were observed in three independent experiments. See also Fig. S4.

Two reports have shown that REDD1 mediates iron chelation-induced mTORC1 inhibition [50, 53]. To verify whether this is a general mechanism, REDD1 protein levels were assessed in Rh30, PCI-13, and A549 cells after 24-h or 4-h CPX or Dp44mT treatment, with/without 1-h FeSO_4_ pretreatment. We found that 24 h iron chelation induced REDD1 expression strongly in Rh30 and PCI-13 cells (Fig. 3a, b), and weakly in A549 cells (Fig. 3c). However, REDD1 was dramatically induced in A549 cells after 4-h CPX or Dp44mT treatment (Fig. 3d). Pretreatment with FeSO_4_ attenuated the induction of REDD1 in all cell lines tested (Fig. 3a–d). A maximal induction of REDD1 protein levels was found in Rh30 and A549 cells following short-time (2–8 h) iron chelating treatment (Figs. 3e, f and S4A, B), suggesting that REDD1 expression is transiently induced by iron chelation.

Since iron chelation-induced hypoxia stabilizes HIF-1α [54, 55], and activated HIF-1 upregulates REDD1 expression, leading to inhibition of mTORC1 [20], next we examined whether the iron chelation-induced transient expression of REDD1 is mediated by HIF-1. For this, a time-course study was conducted. In line with the expression pattern of REDD1, HIF-1α expression was more transient in A549 cells (2–4 h) than that in Rh30 cells (2–12 h) (Figs. 3g, h and S4A, B). After 24-h iron chelation, a considerable level of HIF-1α protein was detectable in Rh30 cells, but a very low or undetectable level of HIF-1α protein was observed in A549 cells (Figs. 3g, h and S4A–D). These results suggest that REDD1 expression is closely related to HIF-1α expression, in response to iron chelation.

To determine the role of the HIF-1-REDD1 pathway in iron chelation-induced mTORC1 inhibition, A549 cells expressing shGFP (control) or shHIF-1α were treated with CPX or Dp44mT for 4 h. HIF-1α knockdown greatly inhibited the increase in REDD1 protein level (Fig. 3i, j). Dephosphorylation in S6K1, but not 4E-BP1, induced by 4-h CPX or Dp44mT treatment, was also attenuated by shHIF-1α (Fig. 3i, j), suggesting a partial contribution of HIF-1/REDD1 to iron chelation-induced mTORC1 inhibition in A549 cells. In Rh30 cells, prolonged HIF-1α and REDD1 induction (Figs. 3f, hand S4A) supports the finding that REDD1 mediates iron chelation-induced mTORC1 inhibition [50, 53]. Taken together, our results suggest that HIF-1/REDD1 is transiently and partially involved in iron chelation-induced mTORC1 inhibition in some cell lines.

### Bnip3 partially and transiently mediates iron chelation-induced mTORC1 inhibition

**Fig. 4:**
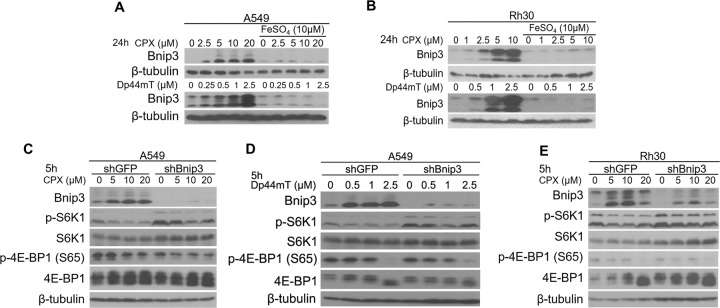
Bnip3 transiently and partially mediates iron chelation-induced mTORC1 inhibition. **a**, **b** A549 or Rh30 cells were pretreated with/without FeSO_4_ (10 μM) for 1 h, and then treated with CPX (0–20 µM) or Dp44mT (0–2.5 µM) for 24 h. **c**–**e** A549 and Rh30 cells, infected with lentiviral shRNA to Bnip3 or GFP (control), were treated with CPX (0–20 μM) or Dp44mT (0–2.5 μM) for 5 h. Cell lysates (**a**–**e**) were subjected to western blotting with indicated antibodies. Similar results were observed in three independent experiments.

In addition to regulating REDD1, HIF-1 also directly regulates the expression of Bnip3 [56], which can negatively regulate Rheb-mTORC1 [22]. Having found that HIF-1 partially mediates iron chelation-induced mTORC1 inhibition (Fig. 3i, j), we tested whether Bnip3 mediates the effect of iron chelation on mTORC1. For this, A549 and Rh30 cells were pretreated with/without FeSO_4_, followed by 24-h CPX or Dp44mT treatment. As expected, the iron chelators induced a robust expression of Bnip3 in the cells, which was blocked by FeSO_4_ pretreatment (Fig. 4a, b), indicating that iron chelation induces Bnip3 expression. Unlike HIF-1α and REDD1 (expressed transiently), Bnip3 was time-dependently induced by iron chelation, and high levels of Bnip3 were detectable after 24-h treatment with CPX or Dp44mT (Fig. S4A, B). Bnip3 knockdown failed to restore long-time (24 h) iron chelation-induced dephosphorylation of S6K1 or 4E-BP1 (Fig. S4E) in A549 cells, but attenuated short-time (5-h) iron chelation-induced dephosphorylation of S6K1, despite no effect on reduced p-4E-BP1 (Figs. 4c–e and S4F). Elevated p-S6K1 was observed in untreated sh-Bnip3 A549 cells compared with untreated shGFP cells (Fig. 4c–e), indicating that Bnip3 indeed negatively regulates mTORC1 under basal conditions. Overall, our findings suggest that Bnip3 partially and transiently mediates iron chelation-induced mTORC1 inhibition.

### AMPK predominantly mediates iron chelation-induced mTORC1 inhibition

**Fig. 5:**
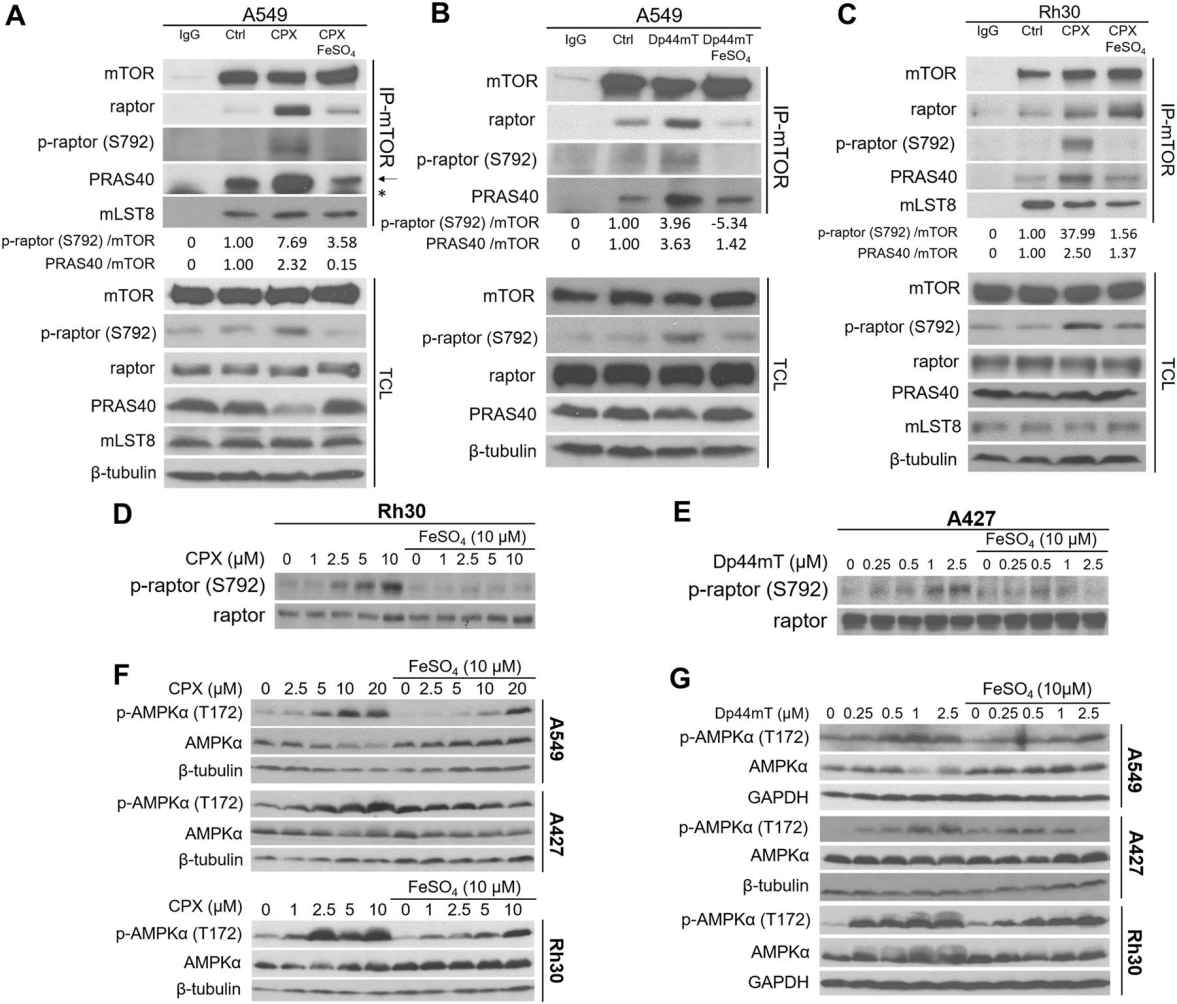
Iron chelation activates AMPK and alters protein interactions in mTORC1. **a**–**c** A549 or Rh30 cells were pretreated with/without FeSO_4_ (10 μM) for 1 h, and then treated with/without CPX (10 µM for Rh30, and 20 µM for A549) or Dp44mT (2.5 µM) for 24 h. Cells were lysed in CHAPS buffer. Cell lysates were incubated with normal goat IgG (negative control), or with goat anti-mTOR antibody to pull down mTOR. Total cell lysates (TCL) and immunoprecipitated products (IP) were subjected to western blotting with indicated antibodies. (Arrow: bands of interest, *non-specific bands). Blots for indicated proteins were semi-quantified using NIH ImageJ. The measurement of normal IgG, considered as the background, was subtracted from other measurements, and defined as 0. Fold changes are shown between the control groups and treated groups (controls were normalized to 1). Similar results were observed in at least two independent experiments. **d**–**g** Indicated cells were pretreated with /without FeSO_4_ for 1 h followed by CPX (0–10 µM or 0–20 µM) or Dp44mT (0–2.5 µM) treatment for 24 h. The whole cell lysates (**d**–**g**) were then subjected to western blot analysis. Similar results were observed in three independent experiments. Related to Fig. S5.

The above results that HIF-1/REDD1 and Bnip3 pathways transiently and partially mediate iron chelation-induced mTORC1 inhibition (Figs. 3, 4 and S4) led us to propose that there may be other important signaling molecules mediating iron chelation-induced mTORC1 inhibition. Due to the consistent inhibition of mTORC1 but not mTORC2 in different cell lines, we first studied whether two mTORC1-specific components, raptor and PRAS40, are affected by iron chelation. Cells were treated with CPX or Dp44mT for 24 h with/without FeSO_4_ pretreatment, followed by co-immunoprecipitation (co-IP). The results showed that treatment with CPX and Dp44mT did not obviously alter the total protein levels of mTORC1 components (mTOR, mLST8 and raptor) in all cell lines tested, but reduced the total protein levels of PRAS40 in A549 cells (Fig. 5a–c). Treatment with the iron chelators consistently increased the binding of PRAS40 to mTOR, which was attenuated by FeSO_4_ pretreatment in all cases (Fig. 5a–c). In A549 and Rh30 cells, CPX and Dp44mT treatment increased the raptor-mTOR interaction (Fig. 5a–c), while in A427 cells, CPX treatment did not enhance the raptor-mTOR interaction, but Dp44mT treatment did (Fig. S5A). FeSO_4_ pretreatment attenuated iron chelation-induced raptor-mTOR interaction in A549 and A427 cells (Figs. 5a, b and S5A), but further enhanced the raptor-mTOR interaction in Rh30 cells (Fig. 5c). These results suggest that the effect of iron chelation on the PRAS40-mTOR interaction is consistent, but its effect on the raptor-mTOR interaction is cell line dependent.

Interestingly, treatment with CPX or Dp44mT consistently increased p-raptor (S792) binding to mTOR in all cell lines tested (Figs. 5a–c and S5A). FeSO_4_ pretreatment consistently attenuated the increased binding of p-raptor (S792) to mTOR (Figs. 5a–c and S5A), suggesting that iron chelation induces p-raptor binding to mTOR. Furthermore, treatment with CPX or Dp44mT also elevated p-raptor (S792) levels in the cells, which was attenuated by FeSO_4_ pretreatment (Fig. 5a–e). Since AMPK phosphorylates raptor on S792, leading to inhibition of mTORC1 [57], next we tested whether iron chelation activates AMPK. As predicted, treatment with CPX or Dp44mT increased p-AMPKα (T172) in a time (Fig. S4A, B) and dose-dependent manner, which was attenuated by FeSO_4_ pretreatment (Fig. 5f, g). Taken together, our findings hint that iron chelation may inhibit mTORC1 by activating AMPK, leading to increased interactions of p-raptor (S792) and PRAS40 with mTOR.

**Fig. 6:**
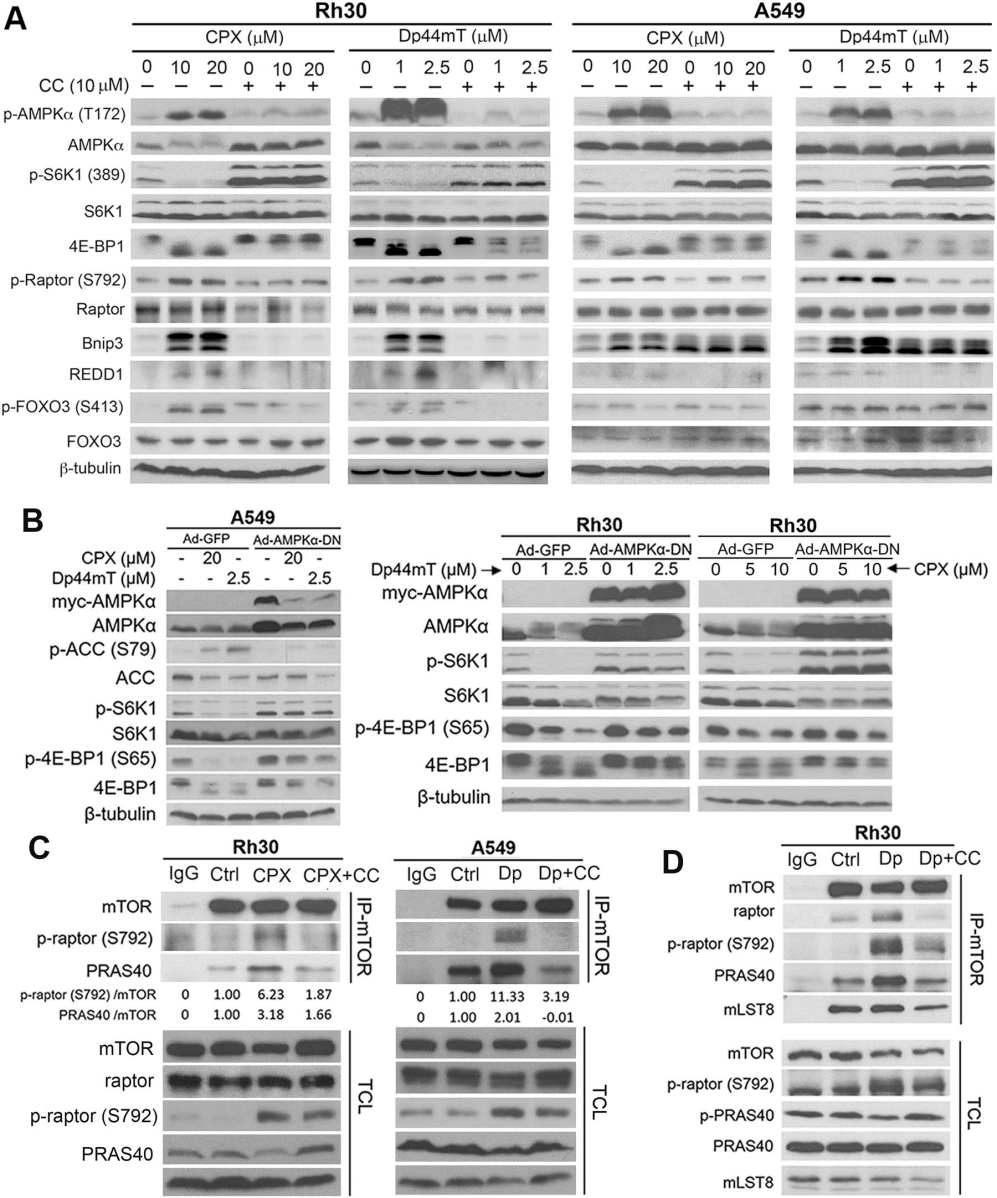
Iron chelation-induced mTORC1 inhibition is partly regulated by AMPK-raptor/PRAS40 pathways. **a** A549 and Rh30 cells were pretreated with/without Compound C (10 µM) for 1 h, and then treated with CPX or Dp44mT at indicated concentrations for 24 h. **b** A549 and Rh30 cells, infected with Ad-GFP (control) or Ad-AMPKα-DN, were treated with CPX or Dp44mT at indicated concentrations for 24 h. (**c** and **d**) A549 and Rh30 cells were pretreated with/without Compound C (10 µM) for 1 h, and then treated with/without CPX (20 µM) or Dp44mT (2.5 µM) for 24 h. The immunoprecipitation and immunoblotting were described in Fig. 5. Similar results were observed in at least two independent experiments.

To determine whether activation of AMPK is truly responsible for iron chelation-induced mTORC1 inhibition, A549, A427, and Rh30 cells were pretreated with Compound C, an AMPK inhibitor, followed by CPX or Dp44mT treatment for 24 h. As expected, Compound C successfully inhibited iron chelation-induced p-AMPKα (T172) (Figs. 6a and S5B, D). Meanwhile, Compound C restored the decreased phosphorylation levels of S6K1 and 4E-BP1 (Figs. 6a and S5D). In addition, ectopic expression of dominant-negative AMPK (AMPK-DN) by infection with a recombinant adenovirus expressing AMPKα-DN [48] successfully attenuated iron chelation-induced dephosphorylation of S6K1 and 4E-BP1 (Fig. 6b). Therefore, our results indicate that AMPK indeed mediates iron chelation-induced mTORC1 inhibition.

To verify whether AMPK mediates increased p-raptor (S792) or PRAS40 binding to mTOR, IP against mTOR was performed after cells were treated with CPX or Dp44mT, with/without Compound C pretreatment. Increased p-raptor (S792) or PRAS40 binding to mTOR was inhibited by Compound C (Figs. 6c, d and S5B, C), suggesting that AMPK mediates iron chelation-increased p-raptor/PRAS40 binding to mTOR.

In addition, NAC pretreatment did not attenuate CPX-induced p-AMPK and p-ACC (Fig. S5E), suggesting that CPX-induced activation of AMPK is independent of ROS induction. This result was consistent with the findings that NAC failed to prevent iron chelation-induced mTORC1 inhibition (Fig. 2a, b).

mLST8 is a component shared by both mTORC1 and mTORC2 [5]. We also tested whether mLST8 was affected by iron chelation. The results showed that iron chelation altered neither total protein level of mLST8 nor the binding of mLST8 with mTOR in cells (Fig. 6d).

**Fig. 7:**
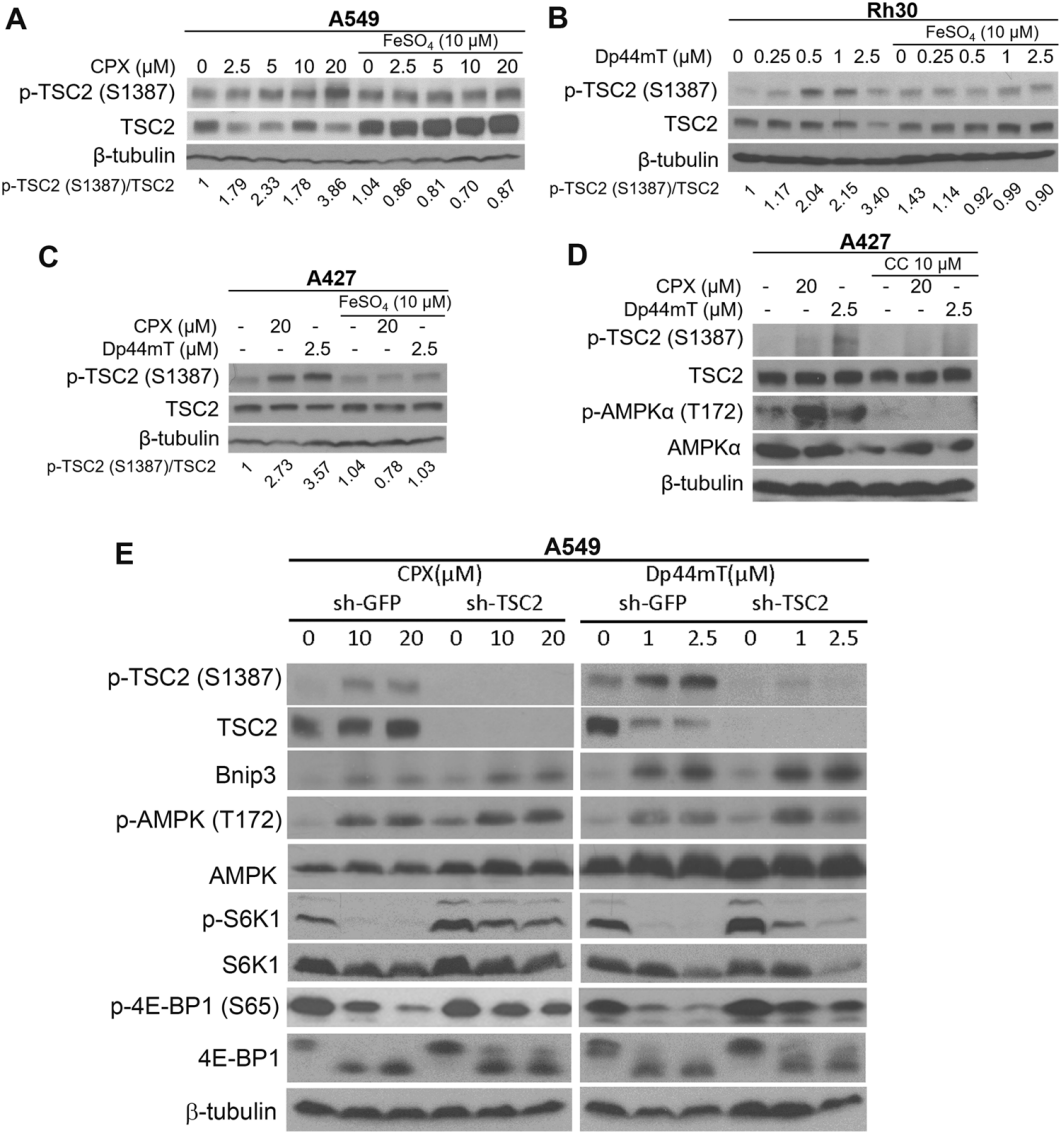
Iron chelation-induced mTORC1 inhibition is partly regulated by the AMPK-TSC pathway. Indicated cells were pretreated with/without FeSO_4_ (10 μM) (**a**–**c**) or Compound C (10 µM) (**d**) for 1 h, and then treated with CPX or Dp44mT at indicated concentrations for 24 h. **e** A549 cells, infected with lentiviral shRNA to TSC2 or GFP (control), were treated with CPX or Dp44mT at indicated concentrations for 24 h. The whole cell lysates (**a**–**e**) were subjected to western blotting with indicated antibodies. Similar results were observed in at least three independent experiments.

Activated AMPK inhibits mTORC1 not only through phosphorylating raptor (S792), but also via phosphorylating and activating TSC2 on multiple sites, including S1387 [24]. Thus, we also determined whether AMPK mediates iron chelation-induced mTORC1 inhibition through TSC1/2. The results showed that 24-h treatment with CPX or Dp44mT increased p-TSC2 (S1387) in A549, A427, or Rh30 cells, which was blocked by 1-h pretreatment with FeSO_4_ (10 μM) or Compound C (10 μM) (Fig. 7a–d), indicating that the increased TSC2 phosphorylation is indeed mediated by AMPK.

To confirm the role of TSC2 in iron chelation-induced mTORC1 inhibition, lentiviral shRNA to TSC2 was used. Knockdown of TSC2 attenuated iron chelation-induced dephosphorylation of S6K1 and 4E-BP1 in A549 cells (Fig. 7e). Collectively, our results suggest that iron chelation-induced mTORC1 inhibition is also mediated via AMPK-induced phosphorylation of TSC2.

Activated TSC1/2 complex inhibits mTORC1 by suppressing Rheb [15]. So, we asked whether iron chelation induces mTORC1 inhibition via inactivating Rheb. For this, HEK293 cells were transiently transfected with plasmids expressing constitutively activated Rheb (Rheb-CA) bearing the Q64L mutation and empty vector (pcDNA3.1), followed by iron chelating treatment. Expression of Rheb-CA fully restored S6K1 phosphorylation, and partially restored 4E-BP1 phosphorylation (Fig. S6A, B), suggesting that iron chelation-induced mTORC1 inhibition is associated with inhibition of Rheb. This further supports that TSC-Rheb axis is involved in iron chelation-induced mTORC1 inhibition.

The molecular mechanism underlying Bnip3-mediated Rheb inhibition remains elusive [22, 58]. We also investigated the effect of double knockdown of Bnip3 and TSC2 on iron chelation-induced mTORC1 inhibition. Because knockdown of Bnip3 partly blocked short-time (5 h), but not long-time (24 h), iron chelation-induced inhibition of p-S6K1 in A549 cells (Figs. 4c, d and S4E, F), sh-Bnip3 and sh-TSC2 A549 cells were treated with CPX or Dp44mT for 5 h. The results indicated that double knockdown of Bnip3 and TSC2 blocked 5-h iron chelation-induced inhibition of mTORC1 more potently than the single knockdown of Bnip3 or TSC2, despite failure to completely restore mTORC1 activity (Fig. S6C). The finding supports that iron chelation inhibits mTORC1 by targeting more other pathways.

## Discussion

In this study, we demonstrated that iron chelation consistently inhibited mTORC1 in multiple cancer cells, but not in normal cells (PCS-201-012, C2C12 and L6) using iron chelators, CPX, Dp44mT, or DFO (Figs. 1e–g and S1). This suggests that cancer cells are more sensitive to iron chelation-induced mTORC1 inhibition than normal cells. In line with this, cancer cells were more sensitive to iron chelation-induced proliferation inhibition than the normal cells (Fig. 1a–d). The results support the notion that iron chelation may be exploited for tumor-selective treatment, and also imply that inhibition of mTORC1 signaling may play a crucial role for the anticancer effect of iron chelation.

Meanwhile, our results revealed that the effect of iron chelation on mTORC2 was cell line dependent, evidenced by western blotting for p-Akt (S473) (Figs. 1h and S2A–C). Similarly, iron chelation on PDK1-mediated p-Akt (T308) was also cell line dependent (Fig. S2D–F). The inhibitory or activating effects of iron chelation on mTORC2/Akt may be related to the diverse genetic backgrounds of various cancer cell lines. Likely, iron chelation-induced p-Akt in some cell lines may result from S6K1-mediated negative feedback on the activity of PI3K [59], or AMPK-mediated activation of mTORC2 signaling [60]. Also, the effect of iron chelation on p-Akt (S473) was time-dependent (Fig. S4F and G). Similar observation has been described in the cells exposed to rapamycin [61]. In this study, we mainly focused on unveiling how iron chelation inhibits mTORC1 signaling. Further research is warranted to unravel how iron chelation impacts the mTORC2/Akt pathway. Identifying the gene(s) or pathway(s) responsible for the differential effects of iron chelation on mTORC2/Akt signaling would be of great importance for the development of efficient combinational and personalized therapy.

Our results showed that iron chelation-induced mTORC1 inhibition is not associated with ROS generation and copper chelation (Fig. 2a–d). Besides, we found that iron chelation by CPX or Dp44mT, at the concentrations that inhibit mTORC1, did not trigger activation of PP2A in Rh30, A427, and A549 cells (Figs. 2e, f and S3A, B), which is in contrast to the observation that PP2A activity is activated by DFO in Caco-2 cells [50]. Whether the discrepancy is due to different cell lines or chelators used remains to be determined. Given that iron is a co-factor for PP2A activity [62], chelating PP2A-bound iron is supposed to inhibit the enzyme activity. Likely, treatment with Dp44mT might have chelated the enzyme-bound iron, leading to inhibition of PP2A (increased p-PP2A) in Rh30 cells (Fig. S3B).

Two studies have described that REDD1 is involved in deferasirox- or DFO-induced mTORC1 inhibition [50, 53]. In line with these reports, here we did observe that treatment with CPX or Dp44mT upregulated REDD1 expression, which was attenuated by FeSO_4_ pretreatment. However, the increased REDD1 expression pattern varied in the cell lines tested (Fig. 3a–f). In addition, consistent with the previous findings that HIF-1 is a transcriptional regulator for REDD1 [63] and Bnip3 [64], we noticed that REDD1 expression was dependent on HIF-1α expression (Fig. 3i, j). Iron chelation-induced expression of REDD1 was transient (peaking at 2–8 h) and well paced with HIF-1α expression, but iron chelation-induced expression of Bnip3 was gradually increased after HIF-1α expression and sustained for at least 24 h (Fig. 3e–h; Fig. S4A and B). However, both HIF-1/REDD1 and Bnip3 pathways only mediated iron chelation-induced inhibition of p-S6K1 but not p-4E-BP1 (Figs. 3i, j, 4c–e, and S4E, F), suggesting that HIF-1/REDD1 and Bnip3 pathways transiently and partially regulate iron chelation-induced inhibition of mTORC1.

In this study, we finally identified that the inhibitory effect of iron chelation on mTORC1 was predominantly mediated by the AMPK pathway. Iron chelation induced phosphorylation of AMPKα (T172) (Figs. 5f, g and S4A, B, F) and its substrates raptor (S792) (Figs. 5a–e, 6a, c, d and S5A–C) and TSC2 (Fig. 7a–e). AMPK inhibitor Compound C or expression of dominant-negative AMPK attenuated iron chelation-induced inhibition of mTORC1 (Figs. 6a, b and Fig. S5D). Iron chelation enhanced the binding of p-raptor (S792) and PRAS40 to mTOR, which was attenuated by FeSO_4_ and Compound C (Figs. 5a–c, 6c, d and S5A–C). These results suggest that AMPK mediates iron chelation-induced mTORC1 inhibition by AMPK-raptor/PRAS40 and AMPK-TSC pathways.

Furthermore, we noticed that inhibition of AMPK with Compound C not only blocked 24-h iron chelation-induced p-raptor (S792) (Fig. 6a) and p-TSC2 (S1387) (Fig. 7c, d), but also suppressed 24-h iron chelation-induced Bnip3 (Fig. 6a) and 5-h iron chelation-induced REDD1 (Fig. S4H). These results suggest that AMPK may also regulate the expression of Bnip3 and REDD1, further supporting that activation of AMPK pathway is a major mechanism for iron chelation-induced inhibition of mTORC1.

We also examined whether the AMPK-FOXO3 pathway contributes to the induction of REDD1 and Bnip3. The results showed that iron chelation remarkably increased the levels of p-FOXO3 (S413) in Rh30 cells, but did not obviously affect the levels of p-FOXO3 (S413) in A549 cells (Fig. 6a). Iron chelation for 24 h induced a robust expression of Bnip3 but a weak expression of REDD1 in both Rh30 and A549 cells. Inhibition of AMPK with compound C suppressed the iron chelation-induced p-FOXO3 (S413), Bnip3 and REDD1 in Rh30 cells (Fig. 6a). These results suggest that the effect of iron chelation on p-FOXO3 (S413) is cell line dependent. In some cases, iron chelation may induce p-FOXO3 (S413), Bnip3, and REDD1 in an AMPK-dependent manner. Likely, the AMPK-FOXO pathway may partly contribute to induction of Bnip3/REDD1 and inhibition of mTORC1. Further research is needed to validate this point.

Bnip3 is required for maintaining metabolic homeostasis by regulating AMPK activity in the liver [65]. Here we observed that Bnip3 knockdown did not alter the levels of p-AMPK (T172), p-Akt (S473), and p-TSC2 (S1387) in A549 cells (Fig. S4F). Further research is needed to understand the differential effects of Bnip3 on the activities of AMPK and Akt in vitro and in vivo.

A new question is that how iron chelation induces activation of AMPK, leading to inhibition of mTORC1. AMPK is a heterotrimeric protein complex, consisting of a catalytic α-subunit and two regulatory β- and γ-subunits, and is regulated both allosterically and posttranslationally [66]. Particularly, AMPKα (T172) can be phosphorylated by at least three kinases, including LKB1 (liver kinase B1) [67–69], CaMKKβ (Ca^2+^/calmodulin-dependent protein kinase kinase β) [70–72], and TAK1 (TGFβ-activated kinase 1) [73], and dephosphorylated by three phosphatases PP2A, PP2C (protein phosphatase 2C) [74], and PPM1E (Mg^2+^-/Mn^2+^-dependent protein phosphatase 1E) [75]. It is known that iron chelation can reduce ATP level (increase AMP/ATP ratio), induce ROS production, and cause DNA damage [40]. LKB1 directly phosphorylates and activates AMPKα (T172) upon energy stress [67–69]. However, we observed activated AMPKα (T172) in A549 and A427 cells (Fig. 5f, g), which are LKB1-null [76], so iron chelation-induced mTORC1 inhibition may be independent of LKB1-AMPK pathway. Clearly, more studies are *required* to address the exact mechanisms underlying iron chelation-induced activation of AMPK and mTORC1 inhibition.

**Fig. 8:**
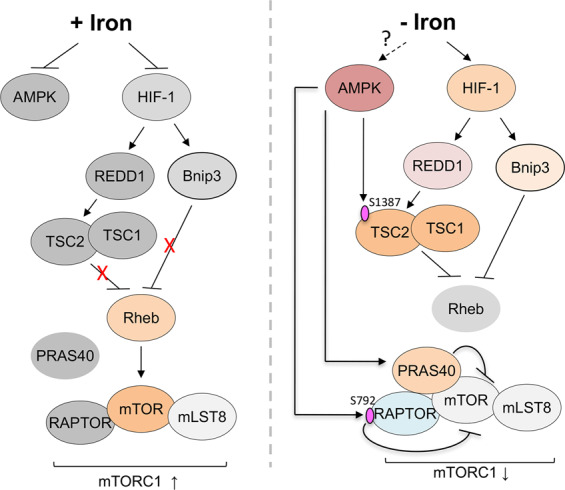
Proposed model of mTORC1 inhibition by iron chelation. When intracellular iron is depleted by iron chelators, AMPK and HIF-1 pathways are activated. On the one hand, activated AMPK phosphorylates raptor (S792) and facilitates the binding of p-raptor (S792) and PRAS40 to mTOR, thereby inhibiting mTORC1. Activated AMPK also phosphorylates TSC2 (S1387) and activates the TSC complex, thus suppressing Rheb-mediated mTORC1. On the other hand, activated HIF-1 upregulates the expression of REDD1 and Bnip3, partially and transiently mediating iron chelation-induced mTORC1 inhibition.

In summary, here we have demonstrated that iron chelation consistently inhibits mTORC1 signaling, but may inhibit or activate mTORC2/Akt in a cell line-dependent manner. Furthermore, we have identified AMPK as a major player mediating iron chelation-induced mTORC1 inhibition (Fig. 8). AMPK mediates iron chelation-induced mTORC1 inhibition through directly enhancing the binding of p-raptor/PRAS40 to mTOR, and by phosphorylating TSC2 (S1387). HIF-1/REDD1 and Bnip3 pathways partially and transiently mediate iron chelation-induced mTORC1 inhibition. Our findings support that iron chelation can be explored for targeted cancer therapy, but further understanding how mTORC2-Akt is activated in tumor cells is warranted for efficient combinational therapy and personalized therapy.

## Materials and methods

### Reagents

Ciclopirox Olamine (CPX) (Sigma-Aldrich, St. Louis, MO) was dissolved in 100% ethanol and stored at −20 °C. Dp44mT (Alfa Aesar, Ward Hill, MA) was dissolved in dimethyl sulfoxide as 100 mM stocks, and stored at −80 °C. Antibodies used are listed in Table S1. Additional materials and methods are available in Online Supplemental “Materials and methods”.

## Supplementary information

Online Supplemental Material

Table S1

Fig.S1

Fig.S2

Fig.S3

Fig.S4

Fig.S5

Fig.S6
