# Supplementary material for: Iron chelation inhibits mTORC1 signaling involving activation of AMPK and REDD1/Bnip3 pathways
Source: Oncogene. 2020 Jun 15;39(29):5201–13. doi: 10.1038/s41388-020-1366-5 (PMC7366895; doi:10.1038/s41388-020-1366-5)
Supplement: Supplementary file 1 — Online Supplemental Material [file 41388_2020_1366_MOESM1_ESM.docx]

**Online supplemental material**

**Supplementary “Materials and Methods”**

**Reagents**

Both iron (II) sulfate heptahydrate and copper (II) sulfate were purchased from Fisher Scientific (Waltham, MA), and freshly prepared to 10 mM and 5 mM stocks with Milli-Q water, respectively, for use. Compound C (EMD Millipore, Billerica, MA) was dissolved in DMSO to prepare a 10 mM stock solution and stored at -20°C. Lipofectamine 2000 Reagent was from Invitrogen (Carlsbad, CA, USA). RPMI 1640, Dulbecco’s Modified Eagle Medium (DMEM), DMEM/F12, and 0.M5% Trypsin-EDTA were purchased from Mediatech (Herndon, VA). Fetal bovine serum (FBS) was from Atlanta Biologicals (Lawrenceville, GA). Type I insulin-like growth factor (IGF-1) (PeproTech, Rocky Hill, NJ) was rehydrated in 0.1 M acetic acid to prepare a stock solution (10 µg/ml), aliquoted and stored at -80°C. All other chemicals were obtained from Sigma (St. Louis, MO, USA), unless specified elsewhere.

**Cell Culture**

Human lung adenocarcinoma (A549 and A427), breast carcinoma (MDA-MB-231), colorectal adenocarcinoma (HT-29), HEK293 and 293T cells were purchased from American Type Culture Collection (ATCC, Manassas, VA). Human head and neck squamous cell carcinoma cells (PCI-13), rhabdomyosarcoma (Rh30 and RD), and Ewing sarcoma (Rh1) cells were described previously (1). Rh30, RD and Rh1 cells were grown in antibiotic-free RPMI 1640 supplemented with 10% FBS, while A549, A427, PCI-13, MDA-MB-231, HT29, HEK293 and 293T cells were grown in antibiotic-free DMEM supplemented with 10% FBS. Human dermal primary fibroblasts (Cat.# PCS-201-012, ATCC) were cultured in Fibroblast Basal Medium supplemented with Fibroblast Growth Kit-low serum (ATCC), and used within 6 passages. All cell lines were cultured in a humidified atmosphere at 37°C with 5% CO_2_. The authentication of each cell line used was not confirmed in this study.

**Lentiviral shRNAs and Adenoviral Infection**

Lentiviral shRNAs to TSC2 and green fluorescence protein (GFP) were produced as described (1). Lentiviral shRNA to Bnip3, HIF-1α, or control was produced using Bnip3 shRNA plasmid (sc-37451-SH, Santa Cruz Biotechnology, Santa Cruz, CA), HIF-1α shRNA plasmid (sc-35561-SH, Santa Cruz Biotechnology), or control shRNA plasmid (sc-108060, Santa Cruz Biotechnology), using the protocol described previously (1). A549 and Rh30 cells were infected with lentiviral shRNA particles in the presence of 8 μg/ml polybrene for 48 h followed by puromycin (1.5~2 μg/ml) selection for 7-14 days. Afterwards, cells expressing the indicated shRNA, as characterized by Western blotting, were used for experiments.

The recombinant adenovirus expressing myc-tagged dominant-negative AMPKα (Ad-AMPKα-DN) and the control adenovirus expressing GFP (Ad-GFP) was described previously (1). Cells were infected with Ad-AMPKα-DN or Ad-GFP (control), as described (1), followed by treatments.

**Plasmid Transfection**

HEK293 cells grown in 100-mm dishes were transfected with the plasmid pcDNA3.1 empty vector or pcDNA3.1 expressing FLAG-tagged constitutively activated Rheb (Rheb-Q64L) (2) (gift from Dr. Yu Jiang, University of Pittsburgh, Pittsburgh, PA), using Lipofectamine 2000 for 24 h, following the supplier’s protocol. Cells were then collected and seeded into 6-well plates for experiments.

**Cell Proliferation Assay**

Cell proliferation was evaluated by cell counting and one solution assay, as described (1).

**Western Blotting**

Cells were seeded in 6-well plates at 5~7 × 10^5^ cells/well according to the size and doubling time of different cell lines. The next day, cells were treated accordingly. Cell lysis and Western blotting were performed as described previously (1). Antibodies used are listed in Table S1. The intensities of indicated bands were semi-quantified using NIH ImageJ software.

**Co-Immunoprecipitation**

Cells (9~10 × 10^6^) were grown in 145-mm dishes according to protein expression levels of different cell lines followed by indicated treatments. Cells were washed twice with PBS on ice, and then collected in CHAPS lysis buffer [40 mM HEPES pH7.4, 120 mM NaCl, 1 mM EDTA, 10 mM pyrophosphate, 10 mM glycerophosphate, 50 mM NaF, 1.5 mM Na_3_VO_4_, 0.3% (w/v) CHAPS, and a cocktail of protease inhibitors (1:1,000, Sigma)]. Following 30-s sonication, samples were centrifuged at 12,000 rpm for 15 min at 4°C. Supernatants were transferred to new tubes. Protein concentration was estimated by NanoDrop 2000C spectrophotometer (Thermo scientific, Waltham, MA). Cell lysates (3.5-5 mg of crude protein) were incubated with 3 µg primary antibodies for 1 h before adding 30 μl of protein A/G agarose beads (sc-2003, Santa Cruz Biotechnology). After overnight incubation at 4°C, samples were centrifuged at 6,000 rpm for 3 min to pellet the beads. Supernatants were aspirated, and the beads were washed with CHAPS lysis buffer for 4 or 5 times, followed by Western blot analysis.

**Statistical Analysis**

GraphPad Prism 5.04 version (GraphPad Software, La Jolla, CA) was used to create the x-y scatter plots or bar graphs with mean ± standard error (S.E.). The statistical analyses were performed using one-way ANOVA (analysis of variance) with the Dunnett post-test or two-way ANOVA with the Bonferroni post-test. *P*-value less than 0.05 was considered to be statistically significant.

**References**

1. Zhou H, Shang C, Wang M, Shen T, Kong L, Yu C, et al. Ciclopirox olamine inhibits mTORC1 signaling by activation of AMPK. Biochem Pharmacol. 2016;116:39–50.
2. Bai X, Ma D, Liu A, Shen X, Wang QJ, Liu Y, et al. Rheb activates mTOR by antagonizing its endogenous inhibitor, FKBP38. Science. 2007;318:977–80.

**Supplementary figure legends**

**Fig. S1.** Iron chelators inhibit mTORC1 consistently. Related to Fig. 1. (A and B) A427 cells were pretreated with/without FeSO_4_ (10 µM) for 1 h, and then treated with CPX or Dp44mT at indicated concentrations for 24 h. (C–F) Indicated cells were serum-starved for 24 h, and then treated with DFO at indicated concentrations for 24 h, followed by stimulation with IGF-1 (10 ng/ml) for 1 h. (G and H) C2C12 and L6 cells were treated with CPX or Dp44mT at indicated concentrations for 24 h. The whole cell lysates were then subjected to Western blotting with indicated antibodies (A–H). Similar results were observed in at least three independent experiments.

**Fig. S2.** Iron chelators inhibit or activate mTORC2 in a cell line-dependent manner. Related to Fig. 1. (A) A427 cells were pretreated with/without FeSO_4_ (10 µM) for 1 h, and then treated with CPX at indicated concentrations for 24 h. (B–F) Indicated cell lines were treated with CPX or Dp44mT at indicated concentrations for 24 h. The whole cell lysates were then subjected to Western blotting with indicated antibodies (A–F). Similar results were observed in at least three independent experiments.

**Fig. S3.** Iron chelation-induced mTORC1 inhibition has no links to PP2A activation. Related to Fig. 2. (A and B) A549 and Rh30 cells were pretreated with/without FeSO_4_ (10 µM) for 1 h, and then treated with CPX (0-20 µM) or Dp44mT (0-2.5 µM) for 24 h. The whole cell lysates were then subjected to Western blotting with indicated antibodies. Similar results were observed in at least three independent experiments.

**Fig. S4.** HIF-1/Bnip3 pathway transiently and partially mediates iron chelation-induced mTORC1 inhibition. Related to Fig. 3 and Fig. 4. (A and B) Rh30 and A549 cells were treated with CPX (10 µM) or Dp44mT (1 µM) for indicated time. (C and D) A549 and Rh30 cells were pretreated with/without FeSO_4_ (10 µM) for 1 h, and then treated with CPX (0-20 µM) for 24 h. (E and F) A549 cells, infected with lentiviral shRNA to Bnip3 or a scrambled sequence (control), were treated with CPX (0-20 µM) or Dp44mT (0-2.5 µM) for indicated time. (G) A549 cells were treated with CPX (0-20 µM) or Dp44mT (0-2.5 µM) for 24 h. The whole cell lysates (A–G) were then subjected to Western blotting with indicated antibodies. Similar results were observed in at least three independent experiments.

**Fig. S5.** AMPK mediates iron chelation-induced mTORC1 inhibition through raptor and PRAS40. Related to Fig. 5 and 6. (A and B) A427 cells were pretreated with/without FeSO4 (10 μM) (A) or Compound C (10 μM) (B) for 1 h, and then treated with/without CPX (20 µM) or Dp44mT (2.5 µM) for 24 h. Cells were lysed in CHAPS buffer and immunoprecipitation (IP) assay was performed to pull down mTOR. Total cell lysates (TCL) and IP products were subjected to Western blotting with indicated antibodies. Blots for indicated protein expressions were semi-quantified using NIH ImageJ. The measurement of normal IgG, considered as the background, was subtracted from other measurements, and defined as 0. Fold changes were shown between the control groups and treated groups (controls were normalized to 1). (C) The protein levels of p-raptor and PRAS40 binding to mTOR in Rh30 cells were semi-quantified by NIH ImageJ. Shown are the mean values ± S.E. (n=2). (D) A427 cells pretreated with/without Compound C (10 µM) for 1 h, and then treated with Dp44mT (0-2.5 µM) for 24 h. (E) Rh30 cells were pretreated with/without NAC (5 mM) for 1 h, and then exposed to CPX (0-20 µM) for 24 h, followed by Western blotting with indicated antibodies.

**Fig. S6.** Iron chelation induces AMPK-mediated TSC2 phosphorylation and inhibits mTORC1 by suppressing Rheb. Related to Fig. 7. (A and B) HEK293 cells were transiently transfected with the empty vector pcDNA3.1 or FLAG-tagged constitutively active Rheb-Q64L (Rheb-CA) plasmid, using Lipofectamine 2000. After transfection for 24 h, the cells were treated with CPX (0-20 μM) or Dp44mT (0-2.5 μM) for 24 h. (C) A549, infected with lentiviral shRNAs to GFP, Bnip3, TSC2, and Bnip3/TSC2, respectively, were treated with CPX or Dp44mT at indicated concentrations for 5 h. The whole cell lysates (A–C) were subjected to Western blotting with indicated antibodies. Similar results were observed in at least three independent experiments.
