# Supplementary material for: Iron chelation inhibits mTORC1 signaling involving activation of AMPK and REDD1/Bnip3 pathways
Source: Oncogene. 2020 Jun 15;39(29):5201–13. doi: 10.1038/s41388-020-1366-5 (PMC7366895; doi:10.1038/s41388-020-1366-5)
Supplement: Supplementary file 2 — Table S1 [file 41388_2020_1366_MOESM2_ESM.docx]

| **Table S1. Antibodies used in this study** | | |
| --- | --- | --- |
| **Antibody** | **Cat #** | **Vender** |
| 4E-BP1 | 9452 | Cell Signaling Technology |
| ACC | 3662 | Cell Signaling Technology |
| Akt1 | sc-5298 | Santa Cruz Biotechnology |
| AMPKα | 5831 | Cell Signaling Technology |
| Bnip3 | sc-56167 | Santa Cruz Biotechnology |
| demethyl-PP2A | sc-13601 | Santa Cruz Biotechnology |
| FLAG | F3165 | Sigma |
| FOXO3 | sc-48348 | Santa Cruz Biotechnology |
| GAPDH | sc-32233 | Santa Cruz Biotechnology |
| HIF-1α | sc-53546 | Santa Cruz Biotechnology |
| mLST8 | A21973 | GenWay Biotech |
| mTOR | 2972 | Cell Signaling Technology |
| mTOR (N-19) (IP) | sc-1549 | Santa Cruz Biotechnology |
| Normal Goat IgG (IP) | sc-2028 | Santa Cruz Biotechnology |
| p-4E-BP1 (S65) | 9451 | Cell Signaling Technology |
| p-4E-BP1 (T37/46) | 2855 | Cell Signaling Technology |
| p-4E-BP1 (T70) | 9455 | Cell Signaling Technology |
| p-ACC (S79) | 3661 | Cell Signaling Technology |
| p-Akt1 (S473) | 4051 | Cell Signaling Technology |
| p-Akt1 (T308) | 9275L | Cell Signaling Technology |
| p-AMPKα (T172) | 2535 | Cell Signaling Technology |
| p-FOXO3 (S413) | 8174S | Cell Signaling Technology |
| PP2Aα | 610556 | BD Transduction Laboratories |
| p-PP2A (Y307) | sc-271903 | Santa Cruz Biotechnology |
| p-raptor (S792) | 2083 | Cell Signaling Technology |
| PRAS40 | 2610 | Cell Signaling Technology |
| p-S6 (S235/236) | 2211 | Cell Signaling Technology |
| p-S6K1 (T389) | 9206 | Cell Signaling Technology |
| p-TSC2 (T1387) | 5584S | Cell Signaling Technology |
| Raptor | sc-81537 | Santa Cruz Biotechnology |
| REDD1 | sc-67051 | Santa Cruz Biotechnology |
| Rheb | 4935 | Cell Signaling Technology |
| S6 | sc-74459 | Santa Cruz Biotechnology |
| S6K1 | 2708S | Cell Signaling Technology |
| TSC2 | 3990 | Cell Signaling Technology |
| β-tubulin | T-8328 | Sigma |
