# Supplementary figures and images for: Iron chelation inhibits mTORC1 signaling involving activation of AMPK and REDD1/Bnip3 pathways
Source: Oncogene. 2020 Jun 15;39(29):5201–13. doi: 10.1038/s41388-020-1366-5 (PMC7366895; doi:10.1038/s41388-020-1366-5)

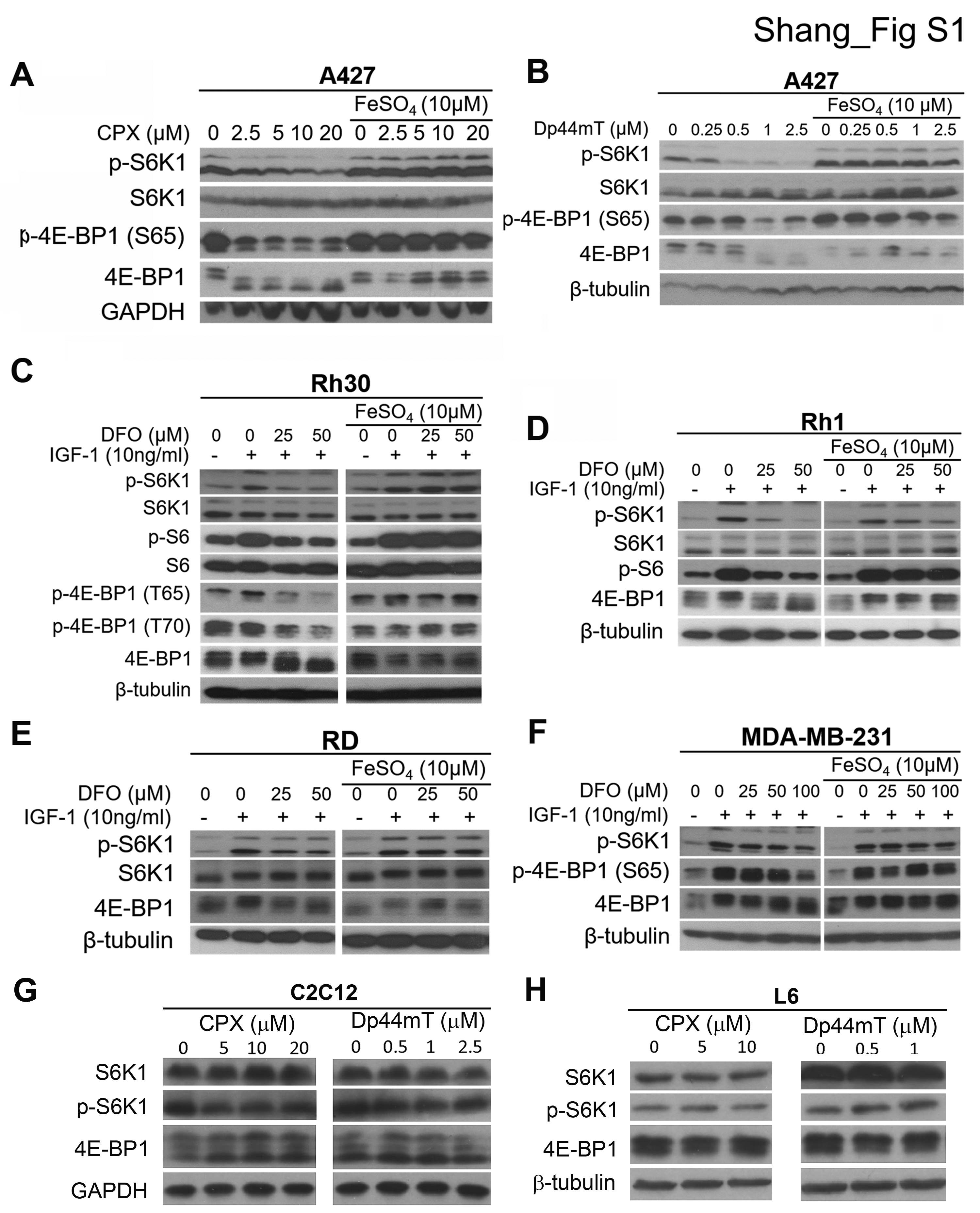

Supplement: Supplementary file 3 — Fig.S1 [file 41388_2020_1366_MOESM3_ESM.tif]

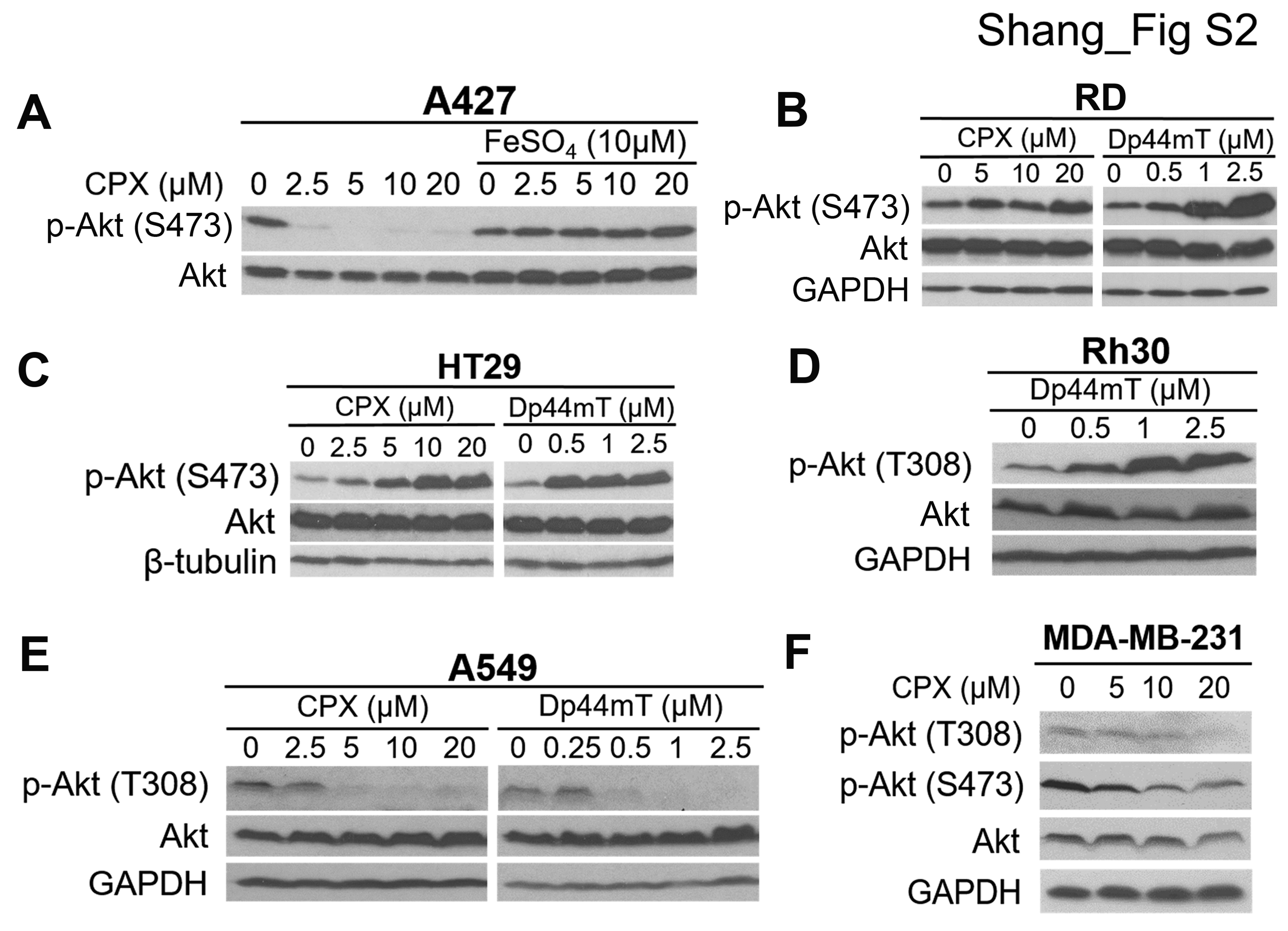

Supplement: Supplementary file 4 — Fig.S2 [file 41388_2020_1366_MOESM4_ESM.tif]

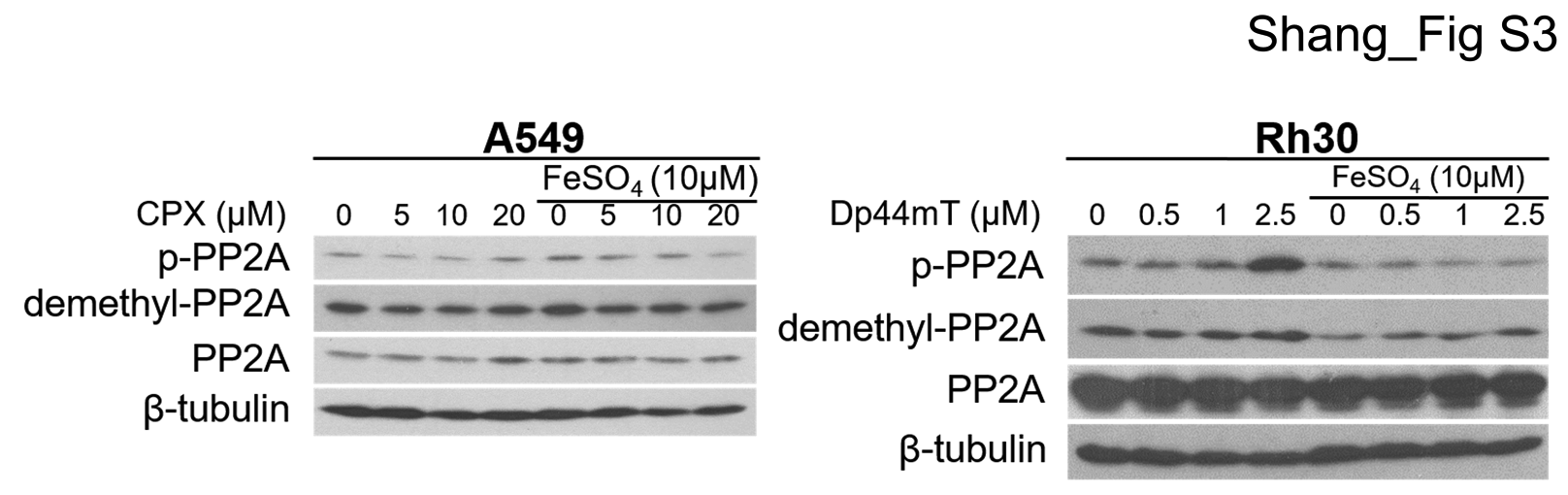

Supplement: Supplementary file 5 — Fig.S3 [file 41388_2020_1366_MOESM5_ESM.tif]

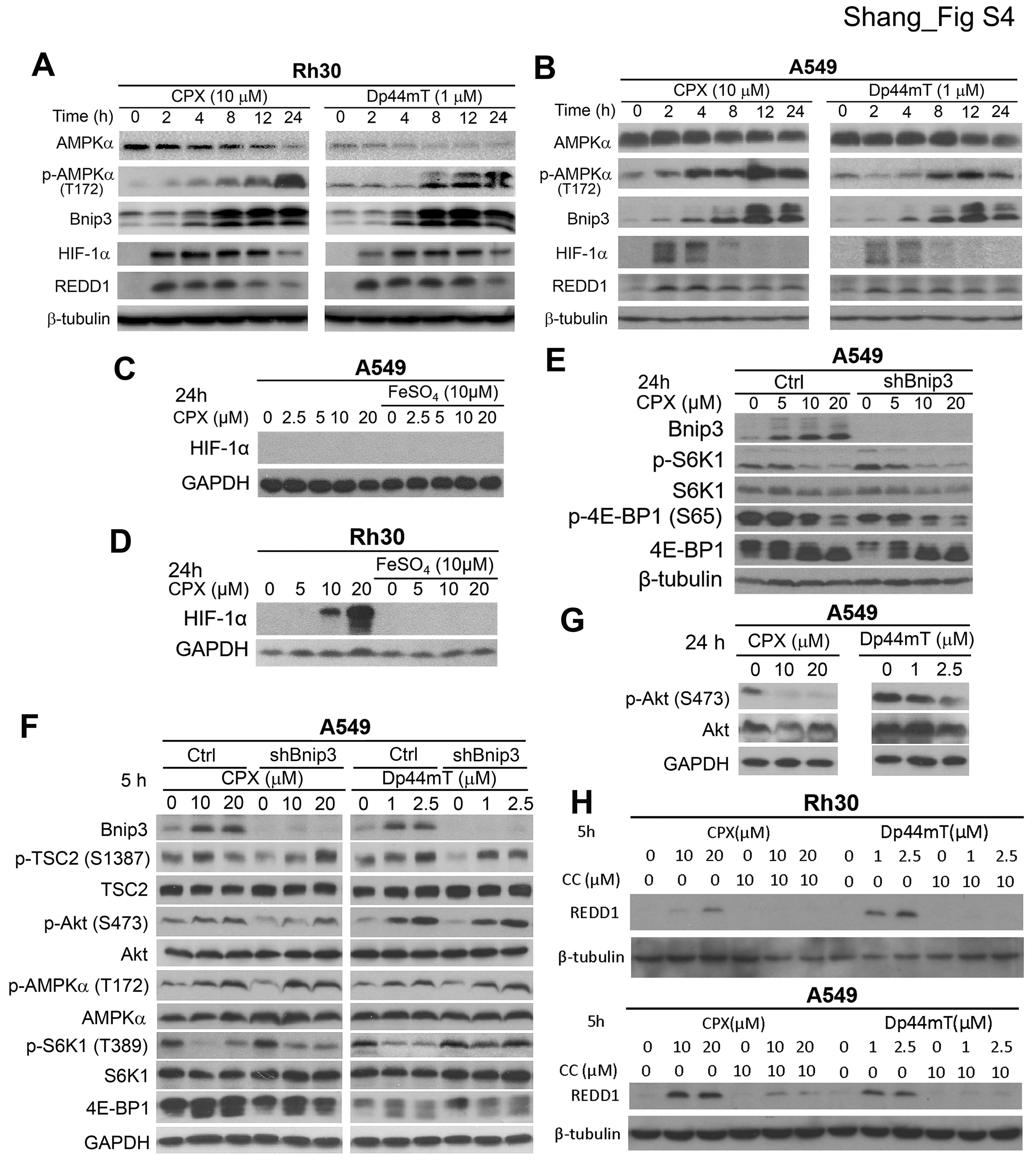

Supplement: Supplementary file 6 — Fig.S4 [file 41388_2020_1366_MOESM6_ESM.tif]

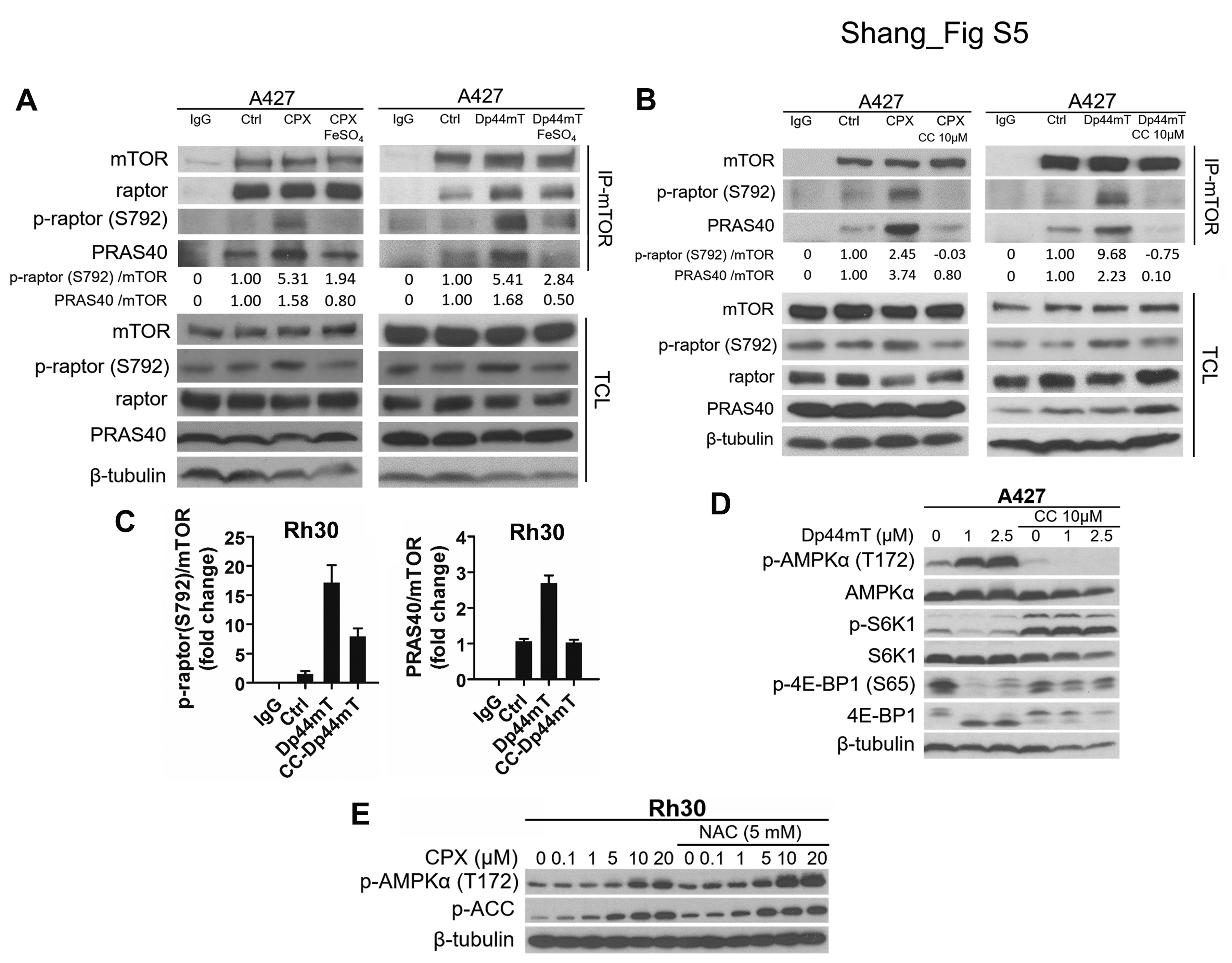

Supplement: Supplementary file 7 — Fig.S5 [file 41388_2020_1366_MOESM7_ESM.tif]

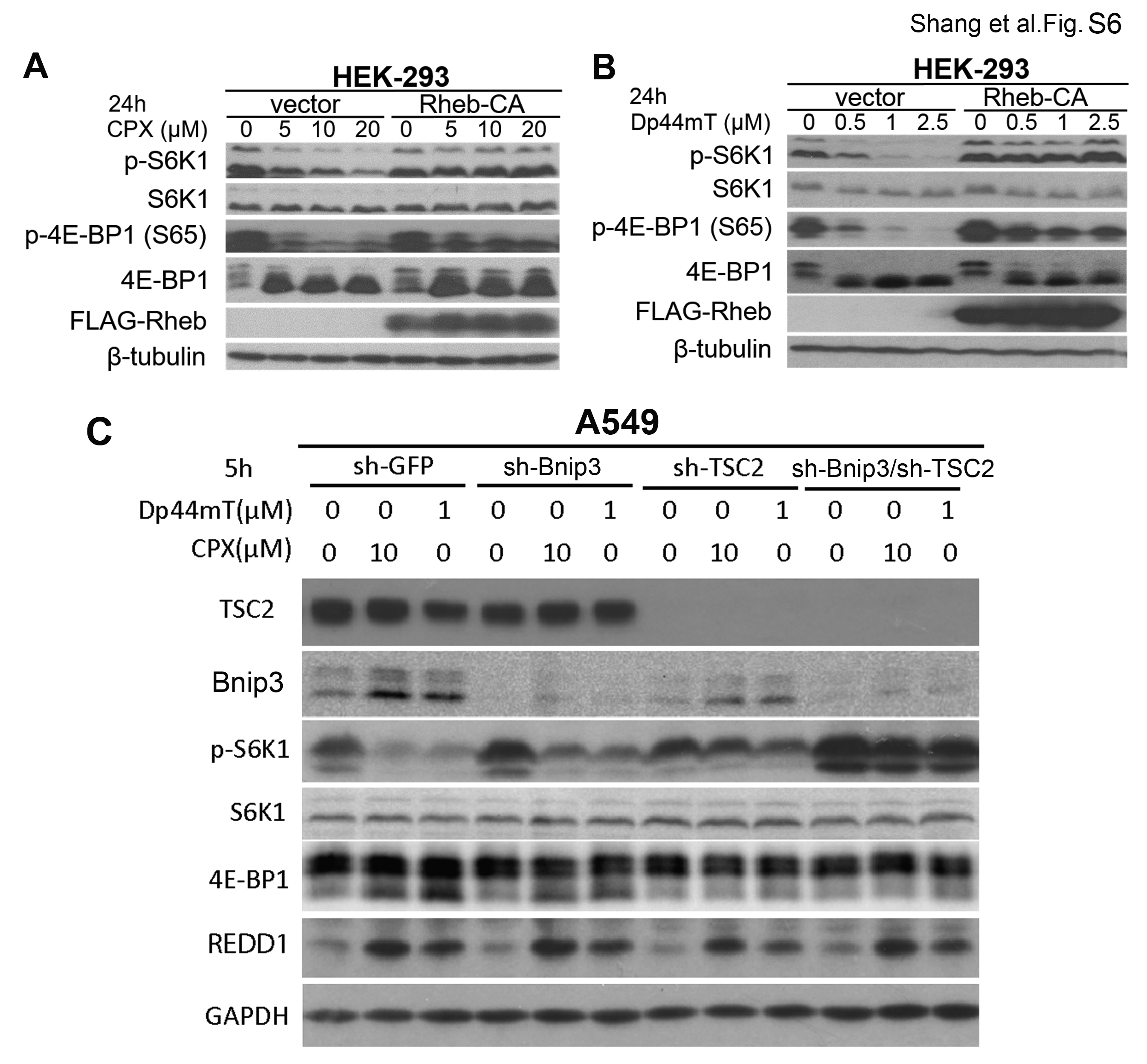

Supplement: Supplementary file 8 — Fig.S6 [file 41388_2020_1366_MOESM8_ESM.tif]
